# Organic Bioelectronics: Diversity of Electronics Along with Biosciences

**DOI:** 10.3390/bios15090587

**Published:** 2025-09-07

**Authors:** Syed Abdul Moiz, Mohammed Saleh Alshaikh, Ahmed N. M. Alahmadi

**Affiliations:** Device Simulation Laboratory, Department of Electrical Engineering, College of Engineering and Architecture, Umm Al-Qura University, Makkah 21955, Saudi Arabia; msshaikh@uqu.edu.sa (M.S.A.); anmahmadi@uqu.edu.sa (A.N.M.A.)

**Keywords:** bioelectronics, organic-bioelectronics, conjugate polymers, electrochemical cell, future perspective of bioelectronics, organic electro-chemical transistor (OECT)

## Abstract

This review article provides an introductory overview of organic bioelectronics, focusing on the creation of electrical devices that use specialized carbon-based semiconducting materials to interact successfully with biological processes. These organic materials demonstrate flexibility, biocompatibility, and the capacity to carry both electrical and ionic impulses, making them an ideal choice for connecting human tissue with electronic technology. The review study examines diverse materials, such as the conductive polymers Poly(3,4-ethylenedioxythiophene) polystyrene sulfonate (PEDOT:PSS) and Polyaniline (PANI), along with critical devices like organic electrochemical transistors (OECTs), which are exceptionally efficient for sensitive biosensing applications. Significant applications include implanted neural interfaces for the brain and nerves, wearable health monitoring, tissue engineering scaffolds that facilitate tissue repair, and sophisticated drug delivery systems. The review acknowledges current challenges, including long-term stability and safety, while envisioning a future where these technologies revolutionize healthcare, human–machine interaction, and environmental monitoring via continuous multidisciplinary innovation.

## 1. Introduction

Over the last forty years, organic electronics has made enormous progress, developing from a niche field of study to a well-known and lucrative technology [1,2,3,4,5]. A great example of a commercially successful use of organic electronics is the use of organic light-emitting diode (OLED)-based screens [6,7,8]. The commercialization of organic photovoltaics, which followed the breakthrough of OLED displays, has shown that organic electronic materials are versatile and feasible for use in renewable energy applications. As research continues, it is anticipated that organic photovoltaic devices will develop and gain broader acceptance in the future [8,9,10,11,12,13,14]. Furthermore, these accomplishments have not only elevated organic electronics to the forefront of consumer electronics, display, and renewable energy, but they have also sparked scientific interest in bioelectronics—the combination of biological systems with organic electronics [15,16,17]. It is well recognized that biological processes may be detected, activated, and even potentially controlled by combining organic electronics with human tissues [18,19].

**Figure 1:**
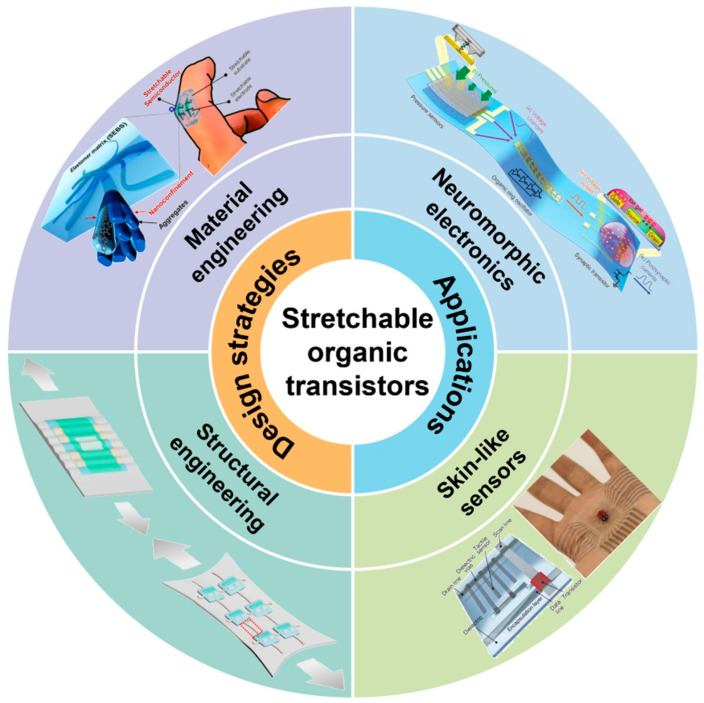
The intersection of material and electronic engineering with respect to application and design strategies for stretchable transistors for organic bioelectronics. Crucial supporting technologies, including the development of stretchy organic transistors for flexible circuits, neuromorphic electrical components for brain-inspired computing, and soft, skin-like sensors. These disciplines seek to develop a novel category of adaptable bionic systems enabling seamless integration between humans and machines. Reprinted from Ref. [26].

The field of organic bioelectronics is significantly progressing towards the creation of seamless, adaptable, and smart interfaces with biological systems [20,21,22,23,24,25]. A fundamental aspect of this advancement is the development of innovative material systems that demonstrate inherent stretchability, biocompatibility, and combined ionic-electronic conduction as shown in Figure 1. These attributes are essential for producing high-performance, flexible organic transistors that can sustain electrical functioning under repeated mechanical deformations, including bending, twisting, and stretching, akin to human skin. The incorporation of these devices into intricate circuits enables neuromorphic electronics, which seek to replicate the structure along with effective processing of biological brain networks for enhanced sensing and localized decision-making. The paramount application of these convergent technologies is the development of multifunctional skin-like sensors—adaptable electronic platforms capable of constantly monitoring a range of physiological indicators (e.g., metabolites, electrolytes, and psychological strain) with high precision. This comprehensive approach, encompassing essential material design to neuromorphic computing and sensing, has the potential to transform closed-loop, autonomous bio-electronic systems capable of diagnosis, therapeutic intervention, and real-time adaptation, signaling into a new era of personalized medicine and human–machine integration [26].

Because of their versatility, simplicity of use with live organisms, and smooth, undisturbed integration with biological systems, electronic devices based on organic/polymer semiconductors are perfect for biomedical applications. This facilitates the development of novel solutions in the fields of healthcare, diagnostics, and brain interface [27]. There are presently several bioorganic materials and products on the market, including cochlear implants designed to restore impaired physiological functions, glucose monitoring for diabetics, and pacemakers/defibrillators [28].

## 2. Diversity of Organic Electronics

The diversity of organic/polymer electronics originates from a broad spectrum of customizable carbon-based semiconducting materials, which may be engineered to be flexible, elastic, biodegradable, or biocompatible [29]. This versatility enables a wide array of applications, including as flexible displays, solar cells, sensors, advanced medical devices, and brain interfaces. The adaptability of this material enables the seamless integration of electronics into innovative forms and functions, beyond the limitations of traditional rigid silicon-based devices [15].

### 2.1. Diversity of Materials

The fundamental advantage of organic electronics lies in its varied and versatile material foundation. This encompasses conductive polymers utilized for electrodes and as well as semiconducting compounds employed in active device components. These materials exhibit significant tunability, allowing for precise engineering of their properties at the molecular level for targeted applications, including flexible displays and biocompatible sensors [30].

### 2.2. Diversity of Functionalities

The capabilities of organic electronic materials rise above just conduction. A significant characteristic is their capacity to be designed for specific functions, making them particularly appropriate for advanced applications. Many materials function as mixed conductors, facilitating the transport of both electrons and ions, which is crucial for interfacing with biological systems such as nerves. Additional materials exhibit electrochromic properties, enabling color change on demand for smart windows, or electroactive characteristics, allowing movement and shapeshifting to function as artificial muscles. Moreover, their chemical properties facilitate the design of (bio)degradable materials, which can be utilized in medical implants that safely dissolve within the body, thereby avoiding the necessity for surgical removal [31,32].

### 2.3. Diversity of Fabrication Techniques

Unlike conventional silicon chips that require production in costly multi-billion-dollar foundries, organic electronics are characterized by their compatibility with low-cost and simple manufacturing techniques [33]. The important advantage is the processability of their solutions; numerous organic semiconductors are soluble, enabling their development into functional inks and processing from fluids at ambient temperature. This enables multiple techniques, such as inkjet and screen printing directly onto surfaces, as well as spin-coating, spray-coating, and vacuum evaporation, to create large-area, thin, and uniform films. These methods facilitate the cost-effective and high-volume fabrication of electronics on lightweight, adaptable polymer substrates, enabling innovative form factors and applications [34,35].

### 2.4. Diversity of Form Factors

The above-mentioned innovative and diverse processing enables the creation of devices that noticeably differ from traditional, rigid structures. Organic electronics can be engineered to demonstrate flexibility and stretchability, allowing for bending, folding, and resistance to compression. Furthermore, they can be produced to be ultra-thin and lightweight, possibly leading to numerous imperceptible bioelectronic applications. That opens the door to totally novel form factors and uses that were not even possible before [35,36].

### 2.5. Diversity of Applications, Specifically in Bioelectronics

Numerous cutting-edge medical applications are made possible by the combination of various organic electronic materials and devices. This includes advanced biosensors for continuous health monitoring, adaptable neural interfaces for safe interaction with the nervous system, and smart implanted devices for precise medication delivery. Furthermore, these technologies are enhancing tissue engineering and advanced prosthetics by providing electrical stimulation for regeneration and enabling realistic movement and feeling [36,37,38].

## 3. Key Aspects of Organic Bioelectronics

Utilizing the unique properties of organic semiconducting materials, organic bioelectronics enables seamless communication with biological processes [39,40,41,42,43,44,45,46,47,48,49,50,51,52]. Here is a list of some of these unique characteristics.

**Figure 2:**
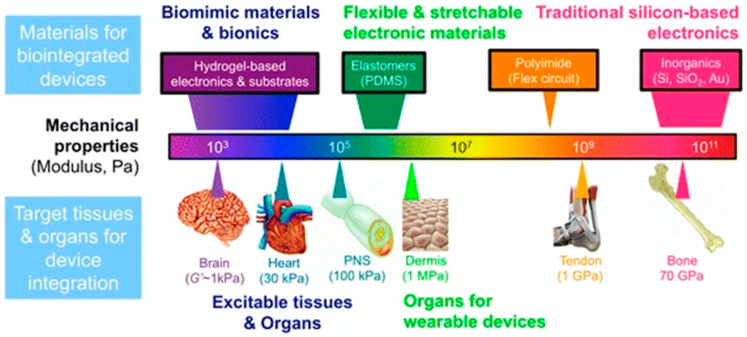
Selection of materials and mechanical characteristics for bioelectronic devices. The chart delineates a repository of common device materials—ranging from pliable hydrogels and elastomers (e.g., POMS, PU) to inflexible inorganics (e.g., Si, Au)—in relation to their Young’s modulus (Pa to GPa). A fundamental design concept emphasizes the need of aligning the mechanical characteristics of the device with those of the target tissue (e.g., brain, skin, bone) to facilitate optimal integration, reduce immunological reaction, and guarantee long-term performance. This framework directs the advancement of several technologies, including adaptable wearables and brain interfaces. Reprinted from Ref. [52].

(1) Biocompatibility: This is the most important characteristic of organic bioelectronics. This is the ability of tiny organic semiconducting compounds/conjugated polymers to interact with biological systems without causing any harm [39,40].
(2) Ionic Conductivity: The ability of organic semiconductors to transport both ionic and electronic currents make them highly suitable for bioelectric applications. Changes in ion flow brought on by biological activity may have a direct impact on the electrical characteristics of organic bioelectronic devices when they encounter cells or tissue [41,42].
(3) Flexibility: Materials used in organic bioelectronics may exhibit conformability, elasticity, and flexibility, which allows them to mirror the mechanical properties of biological tissues [43,44].
(4) Soft Interface: The human body and electronic devices are connected by means of flexible and adaptive interfaces made possible by organic electronics. This reduces the chance of pain or incapacity and improves fitness [45].
(5) Biological Signaling: Bioelectronic devices and living systems may communicate in both directions thanks to the ability of organic electronic materials to transform electrical signaling into cellular signals and vice versa [17,46].
(6) Biological Sensing: This capability, often referred to as biological sensing, is essential for applications such as neural interfaces, which use organic electronics to detect, trigger, and regulate cerebral activity [47,48].
(7) Customizing Functionalities: Organic electronic materials may be used to do several tasks in a single device, such as medication delivery, sensing, and activation [49,50].
(8) Multifunctionality: Multifunctionality makes it possible to develop intelligent, flexible, and integrated bioelectronic systems that can address difficult biological issues [48,51].
(9) Bioelectronic System Applications: It necessitates a thorough assessment of the mechanical characteristics, including Young’s modules, and suitability for living things. Many conducting polymers and natural polymers—organic materials—have excellent biocompatibility, reducing the likelihood of adverse responses or tissue injury, as shown in Figure 2.

Figure 2 illustrates the essential foundation of mechanical compatibility for effective bio integrated devices, correlating various material classes with the mechanical properties of specified biological tissues. The graphic aligns soft, low-modulus organic materials—such as hydrogels, elastomers (e.g., Polydimethylsiloxane (PDMS), Polyurethane (PU)), and conducting polymers (e.g., PEDOT:PSS)—with excitable tissues like the brain, heart, and peripheral nerves (PNS), which display moduli in the kPa range. This consistency is essential for creating conformal, minimally invasive interfaces that reduce foreign body reaction and guarantee long-term signal integrity. In contrast, traditional rigid electronic materials such as silicon, gold, and polyimide (PI) exhibit moduli many orders of magnitude greater (GPa), resulting in a mechanical mismatch that may cause inflammation and device malfunction. This picture highlights the essential function of organic electronic materials as the only category capable of attaining the seamless, biomimetic integration necessary for sophisticated applications in brain interfacing, wearable monitoring, and implantable bioelectronics [52].

## 4. Applications for Organic Bioelectronics

Bioelectricity is an inherent characteristic of all living organisms because charge carrier gradients generate currents and voltages [53]. The fundamental processes that underpin biological activity, such as the activation of voltage-gated Ca+ channels, depend on complicated bioelectrical systems that involve charge exchange. Furthermore, the establishment of communication channels between different organs and tissues depends on these systems [54,55]. Examples of such biological processes include receptor/transporter molecules, ion channels, ion pumps, and gap junctions [56].

**Figure 3:**
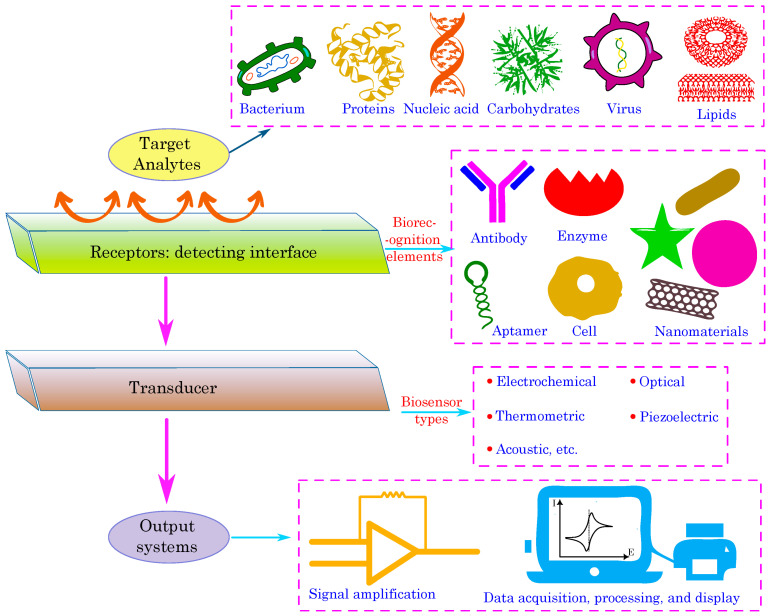
A generalized schematic illustrating the components of a biosensor, emphasizing the significance of organic semiconductors Reprinted from Ref. [57].

Figure 3 illustrates a generalized schematic of a biosensing platform, outlining the essential components from recognition of molecules to signal output. In this design, organic semiconductors serve an essential function, especially as the primary material in the transducer component. Their intrinsic softness and mechanical flexibility provide a flexible interface with biological systems, reducing interfacial stress and enhancing signal fidelity during the detection of biological processes at the receptor layer.

The biocompatibility of several organic semiconductors, including PEDOT:PSS, enables their direct integration into biological output systems such as enzymes and cells, hence enhancing effective biotic-abiotic communication. The diverse electronic and optical characteristics of organic semiconductors make them highly appropriate for various biosensor types, such as electrolyte-gated organic field-effect transistors and organic electrochemical transistors for electronic sensing, as well as active layers in optoelectronic systems for optical detection. Their ability for low-temperature processing and compatibility with flexible substrates facilitates the creation of innovative automated components and wearable designs. Thus, organic semiconductors function as optimal transducer materials, proficiently transforming a biological stimulation into an amplified and processable electrical or optical signal, so connecting biological recognition with data collecting, processing, and presentation.

Three critical steps are involved in the biosensor’s operation as shown in Figure 3: (1) A receptor element that selectively binds to the target analyte at the detection interface; (2) a transducer that transforms the biological recognition event into a quantifiable signal; and (3) signal amplification and data processing devices. Organic semiconductors are especially well-suited for transducer applications, allowing a variety of output systems including optical (e.g., light-emitting diodes, phototransistor or photodetectors), piezoelectric, acoustic, thermometric, and electrochemical readouts. Their intrinsic biocompatibility, adaptability, and potential for economical production render them optimal for developing sensitive and multifunctional biosensing systems [57].

Thus, organic bioelectronic devices have a broad range of intriguing applications, such as

(1) Organic bioelectronic devices improve neurological sensing and stimulation, peri-prosthetics, brain–computer interfaces, drug delivery, and treatments for a range of brain disorders by tracking, promoting, and controlling cerebral (brain) activity [28,58,59].
(2) The flexibility and adaptability of organic bioelectronic devices facilitate the development of soft neural interfaces that could be implanted into the brain and nervous system to improve integration [60].
(3) Organic bioelectronic retina implantation provides sophisticated, compact, non-intrusive devices by using the tunable electrical and optical properties of organic semiconductors. For patients with retinal degenerative illnesses, these devices may interact with the delicate retinal tissue and partially restore vision [61].
(4) Small semiconducting materials are now being investigated by researchers for usage in organic bioelectronic devices for a range of cardiac applications. These include tissue engineering scaffolds that can efficiently monitor and regulate cardiac activity via connections to the heart, pacemakers, and cardiac defibrillators. Furthermore, these devices may be very beneficial for tissue regeneration and repair [62].
(5) Bone tissue engineering would make use of organic bioelectronic materials. These elements have shown potential in increasing bone cell development, activating bone cells, and permitting targeted medication release. This might be useful for the regeneration and repair of damaged or diseased bone structures [63].
(6) Biosensors that are very sensitive and selective may be designed using organic electrical components. Numerous metabolites, physiological signals, and biomarkers may be detected using these biosensors [49].
(7) Continuous, real-time monitoring of health indicators is possible with integrated biosensing devices, which may help with early illness detection and personalized treatment [64].
(8) Organic bioelectronic materials may be active ingredients, substrates, or frameworks in tissue engineering that aid in the growth, differentiation, or regeneration of sick or damaged tissues [65].
(9) It may be possible to create organic bioelectronic devices that would allow the localized release of therapeutic chemicals in response to external triggers or biological signals, allowing for precise and targeted drug delivery [66].
(10) Organic bioelectronic materials may perform a variety of functions, including drug release, actuation, and sensing, all within a single system. This enables the development of tailored and adaptable therapeutic approaches [67].
(11) Adding electrical signaling and stimulation to these organ-based systems may promote tissue growth and repair [68].
(12) Prosthetic limbs, exoskeletons, and various other assistive technologies are examples of organic bioelectrical devices that may improve movement, sensory feedback, and the connection between people and electronics [69].
(13) The compatibility and biomimetic design of organic bioelectronics may enhance these assistive devices’ usability, comfort, and user experience [70,71].

**Figure 4:**
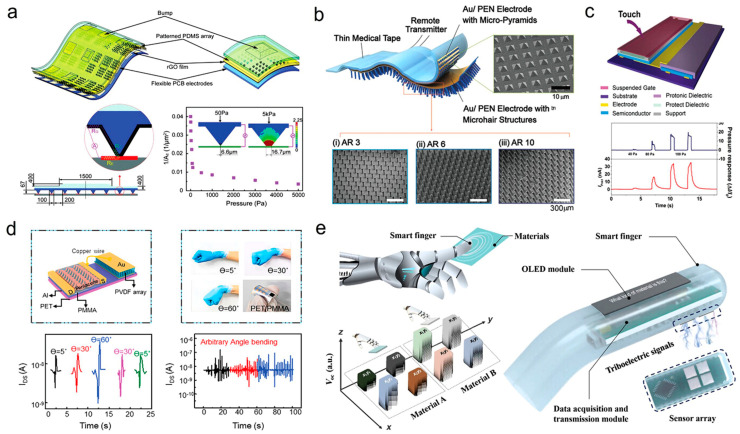
Application of organic electronic devices for bio sensor technology. (**a**) A schematic drawing of a three-dimensional resistive sensor made up of a printed PDMS grid, a rGO film, and bendable PCB wires that shows how the resistance changes with pressure. With permission, this was copied. (**b**) A capacitive pressure sensor made of Au/PEN electrodes combined with micro-pyramid and micro hair structures on a PDMS membrane, which makes it more sensitive and flexible. (**c**) A dual organic transistor device with a capacitive pressure sensor and a synaptic OFET that shows how to recognize touch and how to respond to pressure with different forces. (**d**) A flexible piezoelectric touch sensor was made with a PVDF grid and Au electrodes on a PET/PMMA base. It was tested to see how well it worked at different bending angles and under dynamic strain conditions. (**e**) A triboelectric “smart finger” sensor with an OLED module built in, able to tell the difference between different types of materials and their roughness on the surface using triboelectric signals, along with a module for collecting and sending data. Reprinted from Ref. [72].

As an example Figure 4 shows organic-based force and pressure sensors for human sensory systems that use several materials and device designs to improve sensitivity, flexibility, and multifunctionality. (a) The triaxial resistive sensor uses a patterned PDMS array, rGO sheets, and flexible PCB electrodes. Stressed PDMS protrusions change contact resistance. This design has great sensitivity in low-pressure situations, making it suitable for accurate force detection, but high-pressure stability is difficult. The capacitive sensor (b) uses Au/PEN electrodes with micro-pyramid and micro hair PDMS layers. Hierarchical topologies increase capacitance variation, improving wearable signal responsiveness and conformability. Dual organic transistor systems combine a capacitive pressure sensor with a synaptic OFET for tactile sensing and neuromorphic signal processing (c). This bioinspired technique combines detection and information encoding like mechanoreceptors. PVDF arrays on PET/PMMA substrates make the piezoelectric tactile sensor (d) durable under mechanical deformation. It outputs consistently throughout bending angles. A sensor array and OLED module in the triboelectric “smart finger” (e) enable real-time material type and surface roughness detection via wireless data transfer. These technologies show how organic materials may transform tactile sensors from force detection to multipurpose, interactive electronics [72].

The biological components are hierarchically structured and include tissues and organs (e.g., neurons that transmit electrical signals analogous to electronic circuits), microbial entities (viruses and bacteria), and cellular constituents (antibodies, digestive enzymes, nucleotides, and metabolites such as lactate). Conjugated organic/polymers are essential to this integration because of their flexible, processable, and biocompatible qualities, which allow for dual ion-electron conductance and applications in actuation treatment and soft bio interfaces. Conversely, the electronic domain replicates biological transport of ions and synaptic plasticity with sophisticated devices like memristors and ion pumps, as well as simple components like electrodes and transistors (channel, source, and drain). Neurological interfaces, biosensors, bioelectronic medical practice, and neural computing are examples of biohybrid technologies, which integrate biological and artificial systems via the use of conductive polymers and electronic components. This framework advances our knowledge of bioelectronic integration and aids in the creation of novel therapeutic and diagnostic instruments by using the special qualities of both biological and electronic materials to connect live things with designated equipment [71,72]. A systematic framework for the analysis and classification of advanced functional materials, as outlined in Table 1.

## 5. Organic Semiconducting Materials for Bioelectronics

In organic semiconductors, electric charge is moved through the combination of hopping with space-charge-limited current (SCLC) processes [83,84,85]. The hopping process involves the transfer of charge carriers across molecular sites, whereas the space-charge limited current regimes is the consequence of favorable bulk characteristics and efficient charge injection at electrodes [86,87,88,89,90]. The hopping and SCLC processes are interconnected; they alter hopping dynamics and have an impact on space-charge accumulation. Therefore, doping of organic semiconductors with various molecular or ionic species enhances their mobility, adjusts electrical characteristics like conductance and charge carrier concentration, and modifies the material’s internal energy distribution. Similarly, an electrical signal might be produced by a biological process that alters the doping of organic semiconductor [91,92]. However, a biological response might be triggered by an electrical signal from a device. Organic semiconductors are thus highly suitable for creating novel bioelectronic devices and interactions with biological systems due to their electrical/electronic abilities, flexibility, adaptability, and biocompatibility [62]. Therefore (i) conjugate polymers [93,94,95], (ii) small molecules semiconductor [19,96,97], and (iii) carbon-based materials (e.g., graphene, carbon-nanotubes, etc.) are the typical categories for organic semiconducting materials. All of these materials have well-documented uses in bioelectronic devices [98,99,100].

### 5.1. Conjugate Polymer

A comparative summary of the main characteristics, advantages, drawbacks, and uses of well-known conducting polymers is given in Table 2. As seen, PT typically has the lowest electrical conductivity, while PEDOT has the greatest (up to 500,000 mS·cm^−1^). The majority of polymers have the property of being biocompatible, which qualifies them for use in biomedical applications such as medication delivery, neural prosthetics, and biosensors. The water-soluble versions of PANi and PEDOT:PSS, as well as the water-processable precursor for Poly(p-phenylene vinylene) (PPV), are noteworthy exceptions to the general restriction of low solubility in water [101,102].

Conductive polymeric materials are essential to organic technologies because they facilitate the movement of both ions and electrons. They also serve as a conduit between biological organisms and computer instruments. One of the most adaptable conductive polymers, PEDOT:PSS (poly(3,4-ethylenedioxythiophene) polystyrene sulfonate) has numerous uses in a variety of fields, including organic light emitting diodes [101,102,103], solar cells [104,105,106], sensors [107,108], field effect transistors [109], EMI shielding [110,111], flexible devices [112], wearable devices [113], supercapacitors [114], batteries [115], electrochromic electronics [116], EMI shielding [117,118], catalyst [119], and more. In organic bioelectronics, this material has generated a lot of attention due to its remarkable electrical conductivity, compatibility with living things, and ease of manufacturing. PEDOT:PSS is capable of producing thin films on surfaces by solution-based deposition. This enables the development of flexible, conformal bioelectronic devices that fit into biological tissues quite well. PEDOT:PSS’s strong ionic conductivity enables efficient charge transfer at the bio-electronic interface, which aids in signal transduction and the development of intricate biosensors and brain interfaces [51]. Dijk et al. examined the production and use of PEDOT:PSS-coated platinum conducting electrodes for cerebral stimulation in [102]. Ludwig et al. have shown that a surfactant templated PEDOT films is very suitable for achieving exceptional neuronal recordings, even for up to six weeks [120]. By providing practical answers to persistent bioelectronics issues, PEDOT:PSS is anticipated to significantly impact the next generation of bioelectronic devices. Additional conductive polymers, such as polyaniline (PANI) and polypyrrole (PPy), have been investigated for usage in organic bioelectronic applications in addition to PEDOT:PSS [121,122].

**Table 2:** Electrical Conductivity, Benefits Limitations, and Applications of Conductive Polymers for Bioelectronics: Polypyrrole (PPy), Polyaniline (PANi), Polythiophene (PT), PEDOT, and PPV.

Property	Polypyrrole (PPy)	Polyaniline (PANi)	Polythiophene (PT)	PEDOT	PPV
Electrical Conductivity (mS·cm^−1^)	10^3^–5 × 10^4^	10^2^–10^8^	10^−1^–10^−4^	3 × 10^5^–5 × 10^5^	1–1 × 10^5^
Key Benefits	High conductivity and stability	High stability and conductivity	Good optical properties	High stability and conductivity	Precursor is water-processable
	Biocompatible	Water-soluble	Biocompatible	Water-soluble (doped with PSS)	Strong optical properties
	Strong mechanical properties		Versatile functionality	Biocompatible	High stability
Major Limitations	Fragile, prone to oxidation	Poor plasticity and cell adhesion	Low conductivity and stability	Low mechanical strength	Insoluble in water
	Insoluble in water	Low solubility	Poor solubility		Requires doping for better conductivity
Common Applications	Biosensors	Biosensors	Biosensors	Antioxidants	Biosensors
	Drug delivery	Antioxidants	Food industry	Drug delivery	Light-emitting diodes (LEDs)
	Neural prosthetics	Bioactuators	Tissue engineering	Neural prosthetics	Photovoltaic devices
	Tissue engineering	Food industry		Electrodes	
		Tissue engineering			
References	[123,124,125,126]	[121,127,128]	[129,130,131]	[131,132,133]	[134,135,136]

Polypyrrole [137] is a flexible conductive polymer that may find value in a variety of biological applications. Because of its high electrical conductivity, it can link to electrically active tissues such as the central nervous system and heart muscle [138]. PPy hydrogels and scaffolds facilitate the attachment, development, and specialization of neural cells, hence increasing their usefulness for nerve regeneration and renewal. The electrical stimulation this device provides facilitates the growth of nerve fibers and the transmission of messages between neurons. This makes it crucial for treating neurological disorders and damage to peripheral nerves [139]. The development of functional cardiac patches for the treatment of cardiac failure and coronary artery blockage is aided by PPy’s high electrical conductivity [140]. Because of this substance’s redox activity, physiological parameters may be measured instantly [141].

Polyaniline is another conducting polymer that has been well studied and is suitable for a range of organic electronic and bioelectronic applications due to its exceptional electrical conductivity, simplicity of production, and strong environmental stability [142]. PANI’s redox-active properties enable the construction of electrochemical sensors and transducers, making it an appropriate material for the detection of a wide range of biological analytes, including neurotransmitters, metabolites, and enzymes. The development of a brain electrode array employing PANI that showed a high capacity for storing charge, a low resistance, and the ability to accurately record neural signals from the cerebral cortex of animal models was thoroughly detailed by Guo et al. [143]. Zhu et al. created a sensor by covering a flexible and conductive substrate with a PANI hydrogel that contained glucose oxidase. This polyaniline (PANI)-based glucose se7nsor demonstrated exceptional sensitivity, selectivity, and stability [128].

Polythiophene (PT), a conductive polymer with electrical characteristics that may be changed and compatibility with living organisms, has also attracted attention in the field of biomedical applications [144]. Neural interfaces may be enhanced by polythiophene-based materials. Their ability to improve neural cell attachment, differentiation, and electric stimulation makes them very desirable for application in brain cell tissue engineering and the therapy of brain illnesses [145].

Conductive polymers, including Poly(p-phenylene vinylene) (PPV), Poly(3-hexylthiophene) (P3HT), and several other similar compounds, are widely used in the area of organic bioelectronics. For sensor, bioimaging, and biosensing applications—especially in monitoring and diagnostics within the healthcare sector—they are indispensable instruments [146].

### 5.2. Natural Polymer

Natural polymers have generated a lot of interest in bioelectronic applications due to their intrinsic biocompatibility, flexibility, biodegradability, and capacity to interact with biological systems. Chitosan, collagen, and silk are being studied extensively as polymers because of their potential use in tissue engineering, brain interactions, biosensors, and systems for delivering drugs. This may be explained by their high conductivity, capacity to connect with living cells and tissues, and ability to provide support [71,147,148,149,150].

#### 5.2.1. Chitosan

Chitosan is a biodegradable and organic polymer with special properties that make it perfect for use in organic bio-electronics. Because it may vary to show electrical and ionic conductivity, it is suitable for application in brain interfaces, biosensors, and organic electrochemical transistors [151]. Chitosan’s hydrophilic properties allow it to expand and absorb water, which makes it possible to create bioelectronic devices that are flexible, adaptable, and conforming. The technology’s capacity to adapt to the complicated and unpredictable forms of the human body improves the tool’s interaction with its biological environment. Chitosan might be used as a framework to immobilize a variety of biomolecules, including cells, proteins, and enzymes. This makes it possible to explore applications in biosensors and tissue engineering [152]. Chitosan’s hydroxyl and amine groups aid in the formation of covalent and non-covalent linkages between biomolecules. As a result, a bioactive surface that can interact with biological systems is created. Chitosan’s versatility and several uses make it a suitable material for the development of organic bioelectronics. Reference [153] is very useful for designing integrated bioelectronic devices and tissue-mimicking structures. A glucose biosensor employing extended gate field-effect transistor technology was constructed using chitosan, an intermediate chemical made from MWCNTs, and an indium tin oxide/polyethyleneterephthalate substrates that can detect SnO_2_ [154].

#### 5.2.2. Collagen

Collagen is a protein that exists in nature and is found in the extracellular matrix. It has remarkable biocompatibility and may enhance electrical communication between biological entities and electronic systems. Collagen has special qualities that make it a desirable material for the development of sophisticated brain interfaces, tissue-engineered structures, and other bioelectronic devices that must interact with the human body in an elegant manner. These characteristics include its unique structure, electrical conductivity, and capability to promote cell proliferation and tissue regeneration [155,156].

### 5.3. Silk

Silk, a biomaterial derived from silkworms, is a naturally occurring protein-based polymer that has enormous promise for a variety of bioelectronic applications. Silk’s unique mechanical properties, biocompatibility, and biodegradability make it an attractive material for the development of flexible, adaptable, and implantable bioelectronic systems. These technologies may be used in a variety of sectors, including as brain cell interfaces, tissue engineering, and wearable medical surveillance systems, and they integrate easily with the human body [157].

Due to their distinct structural, electrical, and biocompatible qualities, a number of naturally occurring organic materials, including gelatine [158], cellulose [159], alginate [160], keratin [161], and DNA [162], have been studied for bioelectronic applications in addition to well-known organic/polymers.

### 5.4. Graphene

Researchers have actively examined graphene as a possible material for brain interfaces, biosensors, and scaffolding for tissue engineering, with a particular focus on organic bioelectronics [163]. Graphene’s ability to increase neural cell proliferation and specialization and its improved electrical charge transfer capabilities have enabled high-performance brain electrodes for precise monitoring and activation of cerebral activity [164]. Advanced biosensors have also been designed to detect a variety of substances, including as proteins, neurotransmitters, and metabolites. By adding biomolecules like enzymes or antibodies to the graphene surface, these biosensors achieve high sensitivity and selectivity.

For better glucose biosensing, Alwarappan et al. used enzyme-doped graphene nanosheets [165]. By electrocatalytically reducing oxygen at the GOD (glucose oxidase)-graphic/GC electrode, Wu et al. and others created a new method for glucose detection [166,167]. Table 3 lists a number of conducting polymers, their forms (such as organic electrochemical transistor (OECT), ion sensitive field effect transistor (ISFET), and organic field effect transistor (OFET), and their applications in the biological and sensing fields along with references.

### 5.5. Carbon Nanotube (CNT)

Carbon nanotubes are excellent for the application in organic bioelectronics technologies due to their light weight, high electrical conductivity, broad surface-to-volume ratio, and excellent biocompatibility. When encompassed into organic electronic devices and interfaces, carbon nanotubes may enhance electric charge mobility, signal transmission, and connectivity between electronics and biological systems [211]. This promotes therapeutic technologies [212], stimulation [213], and sensing [214] in the fields of healthcare, neural technology, and regenerative healing [212].

Conducting polymers have been combined with other organic and inorganic elements, such as carbon nanotubes and ceramics, to create sophisticated composite materials with enhanced mechanical, electrical, and biological capabilities. Hybrid systems expand the potential of organic bioelectronics by enabling the creation of very versatile and flexible systems that can easily communicate with biological systems [215].

## 6. Organic Bioelectronic Devices

There are innovative uses for organic bioelectronic devices in the fields of rehabilitation, regenerative medicine, and general medicine. Prescription delivery devices, brain user interfaces, tissue engineering frameworks, and wearable sensors are a few applications.

### 6.1. Core Device Architectures

#### 6.1.1. Organic Electrochemical Transistors (OECTs)

Originally developed by White’s team in 1984, the organic thin film transistor is also known as the organic electrochemical transistor. Because of its remarkable water stability, it has lately attracted attention and is appropriate for use in biological sciences [216]. Because OECTs effectively integrate electrical and ionic transport, they are finding growing use in bioelectronics as well as other organic devices. Organic material having mixed ionic-electronic conductivity is used as the channel material in OECTs, enabling ion doping throughout the channel. The enhanced transconductance and signal-to-noise ratios of these devices make them ideal for biosensing and capturing electrophysiological signals. The way the tools function and the setting in which they may be employed are significantly influenced by their attributes [44].

Conducting organic/polymer’s channel conductivity is regulated by an organic electrochemical transistor via electrochemical interactions. The semiconductor of the OECT device serves as the channel and is often made of conjugated organic/polymers (e.g., PEDOT:PSS). A non-polarizable Ag/AgCl or similar kind of electrode is integrated into a gate electrode that is interacting with an electrolyte solution [176,217,218,219]. Typically, an organic field-effect transistor regulates the current flow through the channel. Ion passage may alter the conductivity of this channel material because of its electrochemically active architecture. Consequently, when a voltage (V_GS_) is supplied, the electrochemical process alters the channel’s electrical I_DS_ by varying the amount of doping at the boundary between the channel and the dielectric barrier [201,220]. Many organic semiconductors, such as conducting polymers or redox-active organic molecules, serve as the active channel in organic electrochemical transistors conductivity [221]. The benefits of OECT may be advantageous for bioelectronic applications due to their ability to convert impulses from cells into electrical signals [222].

The function of OECT is determined by the interplay of charge and ion transport, which modulates the current (I_DS_) flowing through the conducting channel from the source to the drain. The transconductance, see Equation (1), can be define as gm = δID/δVG and can be used to assesses the amplification properties of OECTs. Hence it enhances the sensing performance, which is defined by the detection limit as the signal-to-noise ratio should be at least larger than 3 [223].

(1) $$g_{m}=\frac{\partial I_{D}}{\partial V_{G}}=\frac{Wd}{L}\mu_{n}C*\left(V_{th}-V_{G}\right)$$

The active layer’s carrier mobility, volume capacitance, and OECT threshold voltage are represented by m, C*, and Vth in the equation above, while W, L, and d stand for the channel’s width, length, and thickness, respectively.

OECTs’ gate, channel, and electrolytes have been functionally modified to increase their transconductance (g_m_) sensitivity and usefulness in detecting proteins, ions [224], neurotransmitters [225], metabolites [2,158,226,227], and nucleic acids [183,228]. Scheiblin and his colleagues have shown that OECTs can be used well for pH sensing [229]. Trans-conductance, a critical measure for evaluating the effectiveness of OECTs, is determined by the volumetric capacitance (C*) and carrier mobility (μn) of Organic Mixed Ionic Electronic Conductors (OMIECs). OMIECs with high μC* values have garnered a lot of interest as a result. OMIECs are perfect for future usage in implantable and wearable bioelectronics because of their numerous desirable qualities, including their capacity to stretch, repair themselves, adhere to biological surfaces, and many other similar qualities.

**Figure 5:**
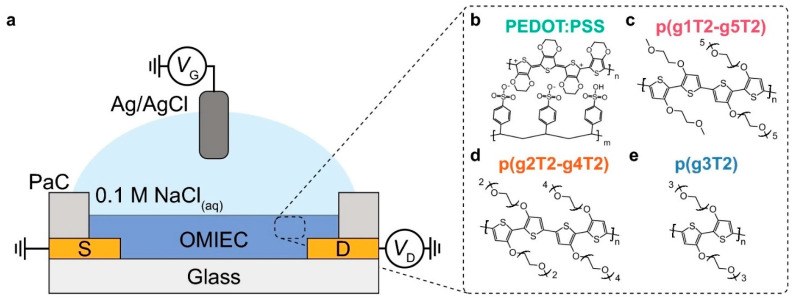
An organic electrochemical transistor’s (OECT) working mechanism. Through electrochemically doping or dedoping the organic mixed ionic-electronic conductor (OMIEC) channel, a gate voltage (VG) delivered via an Ag/AgCl reference electrode in a 0.1 M NaCl electrolyte modifies the drain current (ID). By injecting or expelling hydrated ions, such as Na^+^, into the bulk polymer film, this reversible technique modifies the hole carrier density and electrical conductivity of the film. The density of ethylene glycol chains on either side that promote ion transport and swelling is crucial for the OMIEC’s volumetric capacitance (C*) and mobility of charge carriers (μ), which in turn control the device performance, which is measured by its transconductance (gm). The copolymers p(g2T2-g4T2), p(g1T2-g5T2), and the standard OMIEC PEDOT:PSS are used to assess the performance of the homopolymer p(g3T2). Reprinted from Ref. [230]. Stability testing of OECT channel materials. (**a**) Schematic drawing of an OECT structure. (**b**–**e**) Chemical structures of the studied channel OECT channel materials (**b**) PEDOT:PSS, (**c**) p(g1T2-g5T2), (**d**) p(g2T2-g4T2), and (**e**) p(g3T2).

Figure 5 shows a working of OECT in little detail as a case study. Whereby electrochemically regulating the doping state of the organic mixed ionic-electronic conductor (OMIEC) as a channel, a gate voltage (VG) delivered via a stable Ag/AgCl reference electrode regulates the current (ID) between the source and drain electrodes. This schematic demonstrates the basic working principle of an OECT. The reversible insertion of hydrated ions (such as Na⁺ from the 0.1 M NaCl electrolyte) into the bulk of the OMIEC film—a procedure known as electrochemical doping—is essential to the thermodynamics of this control. By compensating for electronic charges on the polymer backbone, this ion ingress (controlled by the electrolyte/OMIEC interface capacitance) increases the hole carrier density and electronic conductivity (for a p-type OMIEC such as PEDOT:PSS) in a depleting operation, or dedoping it to decrease conductivity. The main physical concept is that the density of ethylene glycol side chains directly determines the polymer film’s ionic permeability and volumetric capacitance (C), which in turn regulates the transduction efficiency (i.e., the transconductance, gm ∝ μC) by affecting both electronic mobility (μ) through modifications to the semiconductor backbone’s microstructure and energetic disorder and ionic mobility (i.e., by facilitating ion transport and swelling) [230].

Table 4 illustrates the diverse array of attributes attainable via OMIEC materials, which we used to evaluate the performance of our OECT devices. This list is not exhaustive; nonetheless, it includes some high-performing polymers such as P(gTDPPT) and PT2gT. These materials surpass others by many orders of magnitude, with normalized transconductance (g_m,norm) surpassing 250 S cm^−1^ and mobility-capacitance (μC*) products nearing 300 F cm^−1^ V^−1^ s^−1^. This comparison approach allows us to objectively assess our channel contents against the state-of-the-art and demonstrate their superiority in certain domains.

#### 6.1.2. Organic Electrochemical Sensors

The biosensors industry is now valued at hundreds of billions of dollars all over the world. Significant growth in this area is being driven by developments in organic bioelectronics and their applications in manufacturing processes, environmental monitoring, healthcare, and other fields [91]. One kind of biosensing instrument that connects biological systems is an organic bioelectronic sensor, which uses organic electronic materials and designs. Since these sensors have unique benefits over traditional inorganic semiconductor-based sensors, they are useful for a wide range of biological and environmental monitoring applications. In order to detect many biomarkers, Sun et al. created ultrasensitive protein sensors by combining pillar [5]arene-COOH (DMP[5]-COOH) with PDBT-co-TT [248].

The OECTs, which take use of organic semiconductors’ ability to convert ions into electrons, are the basic building blocks of organic electrochemical sensors. An OECT-based sensor interacts with the organic semiconducting material in order to regulate the electrical characteristics of the transistor, like voltage or current, using an analyte, such as a particular molecule or ion. Finding the target analyte’s existence or concentration will aid in the identification and correlation. Organic bioelectronic sensors may be categorized in a number of ways. A few of them are listed below.

(1) Enzymatic biosensor, using enzymes as the biological recognition component, enzymatic biosensors detect and measure certain substances, such as glucose [249], urea [250], or cholesterol [251].
(2) Immunosensors, biosensors that identify and measure certain biomarkers or antigens using antibodies or antibody fragments [252].
(3) DNA/Geno sensors, also known as biosensors, use nucleic acid probes, such as DNA or RNA, to monitor genetic sequences or mutations [253].
(4) Entire-cell biosensors use entire living cells, such as bacteria, algae, or mammalian cells, as the biological component to detect a variety of analytes or environmental changes [254].
(5) Tissue-based biosensors detect and measure toxins, medications, or other compounds using slices or cultures of biological cells, such as muscle or liver [255].
(6) Aptamer-based biosensors employ single-stranded DNA or RNA aptamers as the identification component to accurately and highly specifically detect various target substances [256].
(7) Microbial biosensors use microorganisms, such as bacteria or yeast, to detect and respond to certain environmental conditions or the existence of target substances [257].
(8) Photonic biosensors: These biosensors use optical methods, such as surface plasmon resonance or fluorescence, to identify and measure biomolecular interactions [258].
(9) Using electrochemical methods such as amperometry, potentiometry, or impedance spectroscopy, biosensors detect and quantify targeted analytes [259,260].
(10) Using the piezoelectric characteristics of materials like polymer films or quartz crystals, piezoelectric biosensors detect and quantify mass changes brought on by biomolecular interactions [261].

#### 6.1.3. Organic Light-Emitting Diodes (OLEDs) for Optogenetics

The solid-state lighting technology, known as organic light-emitting diodes, can produce light that is precisely localized. This makes them suitable for optogenetic applications. The approach of optogenetics involves using light-sensitive proteins, such as channel rhodopsins, to change the behavior of certain cells or tissues in living creatures [262]. The contraction of muscles may be induced by connecting an OLED display to a nerve terminal of the gracilis muscle in the hindlimb [263].

**Figure 6:**
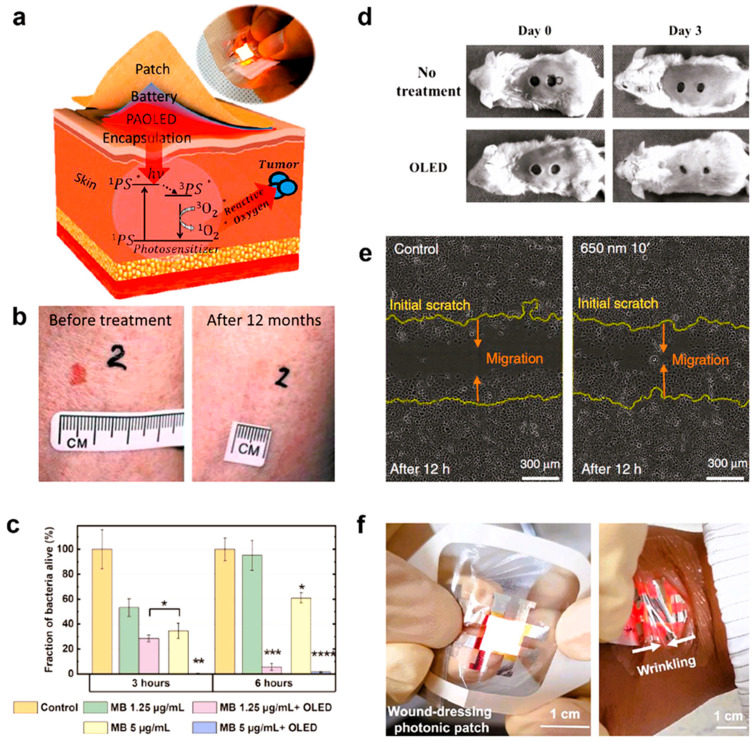
Illustrates phototherapeutic devices that are based on flexible OLEDs. (**a**) Illustration of a wearable OLED patch for photodynamic therapy (PDT). The patch activates a photosensitizer (PS) that is applied to the epidermis. Upon light absorption, the PS generates cytotoxic singlet oxygen (^1^O_2_) to eliminate tumor cells. (**b**) Clinical photographs that illustrate the successful therapy of a basal cell carcinoma lesion on a patient’s nostril with OLED-PDT over a 12-month period. (**c**) The effectiveness of antimicrobial photodynamic therapy (PDT) is evaluated by using methylene blue (MB) as a photosensitizer. A significant reduction in bacterial survival is observed only when using OLED illumination. Statistical significance compared to control (* *p* < 0.05, ** *p* < 0.01, *** *p* < 0.001, **** *p* < 0.0001). (**d**) In vivo evidence of accelerated lesion healing in mice following photobiomodulation (PBM) treatment administered via an OLED device. (**e**) An in vitro scratch experiment that illustrates the enhanced migration of human keratinocyte cells under 650 nm OLED light, a critical mechanism in wound repair. (**f**) A transparent wound dressing is depicted in a photograph, showcasing a flexible, ultra-thin OLED that is incorporated within it. This design underscores the potential for adaptive and wearable medical applications. Reprinted from Ref. [264].

Figure 6 shows all the different ways that OLED technology can be used in phototherapeutic treatments. As shown in panel a, wearable OLED patches provide a new way to do radiation therapy. In this therapy, light from the device excites a photosensitizer that is applied on the skin or embedded in the tissue. This causes the production of damaging singlet oxygen (^3^O_2_) in the targeted death of cancer cells. The practical effectiveness of this method is strongly supported by Figure 6b, which shows that a basal cell cancer tumor went away after being treated with a red-emitting OLED. In addition to its use in cancer, OLED has a lot of promise as an antibiotic. Figure 6c shows how methylene blue, when paired with OLED lighting, can kill germs very effectively, depending on the amount and intensity of light used. Also, OLEDs are very good at photo-biomodulation, which speeds up the healing process. Figure 6d shows how OLED treatment speeds up wound healing in an animal model. This is also seen in Figure 6e, which shows how OLED light at 650 nm greatly increased the movement of keratinocytes, a key type of cell in wound repair. Lastly, Figure 6f shows how this technology can be used in real life. It shows an ultra-thin, bendable OLED that is perfectly bonded onto a clear wound patch. This shows how photonic medical devices could be made to fit your body and be worn [264].

In optogenetics, OLEDs provide spatial control by enabling the customization of their shapes and sizes to precisely target regions or cell concentrations inside a sample or organism. Because OLEDs have a high switching speed, light emission may be precisely timed, giving temporal control. Effective optical stimulation of photosensitive proteins is facilitated by this characteristic. Multimodal integration refers to the ability of OLEDs to be coupled with other functional elements, such electrodes or sensors. This combination makes it possible to utilize visual stimulation and electrical/chemical recordings from the selected cells or tissues at the same time. When organic materials used in OLEDs are designed to be biocompatible, the possibility of adverse consequences upon implantation or when they are connected to biological systems is decreased. OLEDs are essential for reducing power consumption and heat production in in vivo optogenetic systems because they can achieve very high power efficiency [265,266].

Empirical research has shown that organic light-emitting diodes may be used in a variety of optogenetic settings. Using organic light-emitting diodes, researchers have effectively regulated neuronal activity by optically stimulating neurons in preparations of brain slices and cell cultures [267,268]. Organic light-emitting diodes are also used in optogenetic devices to control neuron activity and behavior in rats and zebrafish [269,270]. Additionally, OLEDs are used in optogenetic regulation of gene expression, which allows for dynamic pathway change [271]. Optogenetic devices based on integrated OLEDs have greatly advanced this technique. These technologies enable cordless and less invasive optical stimulation, which opens up new possibilities for in vivo applications and potential therapeutic treatments [264,272]. Along with improvements in optogenetic methods and materials, OLED technological advancements should expand the potential of OLED-based optogenetics in future therapeutic treatments.

#### 6.1.4. Stimulus and Organic Bioelectronic Actuators and Generators

In order to convert electrical signals into mechanical movement or deformation, organic bioelectronic systems rely heavily on organic actuators. This enables the devices to react to biological systems in a dynamic and intelligent manner. Hydrogels and other bioactuators are made of organic materials that may be controlled by external stimuli. These stimuli include changes in light, humidity, pH, redox state, current, magnetic fields, and temperature. By drastically altering volume or phase, the bio actuators may exhibit reversible swelling or shrinking behavior when these stimuli are applied and modified [42,273]. Organic actuators have inherent structural flexibility and biocompatibility, making them ideal for integration with smooth, dynamic biological tissues. This aids in the creation of intricate interfaces for a variety of applications, such as active implantable devices [274], microfluidic valves [275], and artificial muscles [276,277]. Additionally, the advancement of intelligent and flexible organic bioelectronic devices is aided by the ability to alter the physical characteristics and responsiveness of organic actuators through material manipulation and molecular configuration, which increases their efficiency and incorporation into biological systems.

To create pulse trains that replicate the nervous system’s function, the Bao research team constructed a signal conditioning circuit system on a skin-like patch. With a driving voltage of less than ±5 V, this patch could represent the typical sensory process [278].

Bao and associates used soft, elastic hydrogel to develop a microelectronic device. They achieved this by replacing the ionic liquid component of PEDOT:PSS with an elastic fluorinated photoresist and used water as the passivation layer. In a mouse model, the micropatterned conducting hydrogel electrode exhibited little inflammatory tissue reaction and mechanical properties suitable with the nerve, with a Young’s modulus of 32 kPa [279].

### 6.2. Functional Materia Plateform

#### 6.2.1. Organic Hydrogels

For the advancement of bioelectronics, the role of organic hydrogels is crucial. Water-filled polymer networks compose these flexible materials, mimicking the extracellular matrix of living organisms [42]. For some desirable properties, organic hydrogels are very compatible with organic bioelectronic systems. These qualities include electrical conductivity, mechanical adaptability, biocompatibility, responsiveness to stimuli, and compatibility with water- and light-based processes [280].

There are several ways that hydrogel might help bioelectronics progress. Hydrogels are also a good match for implanted bioelectronic devices since they are biocompatible and conform to biological tissues [281]. Their ability to absorb and store water facilitates the efficient movement of ions at the interface between tissues and devices and produces electrochemical signals. Certain hydrogels have beneficial electrical qualities that aid in signal transduction, such as high ionic conductivity. These materials have a lot of promise for usage in bioelectronic devices in the future due to their remarkable electrical characteristics and flexible, adaptable nature [282,283].

The injectable conductive polymer hydrogels are a novel class of biomaterials with enormous promise for use in organic bioelectronic applications. Injectable polymer-based hydrogels have considerable potential for treating various tissue ailments, such as injury to the heart, nerves, and skeletal muscles [284,285,286]. Most hydrogels are made from water-friendly polymers that can conduct electricity, like PEDOT:PSS, polyaniline, polypyrrole, or a type of polythiophene [287].

**Figure 7:**
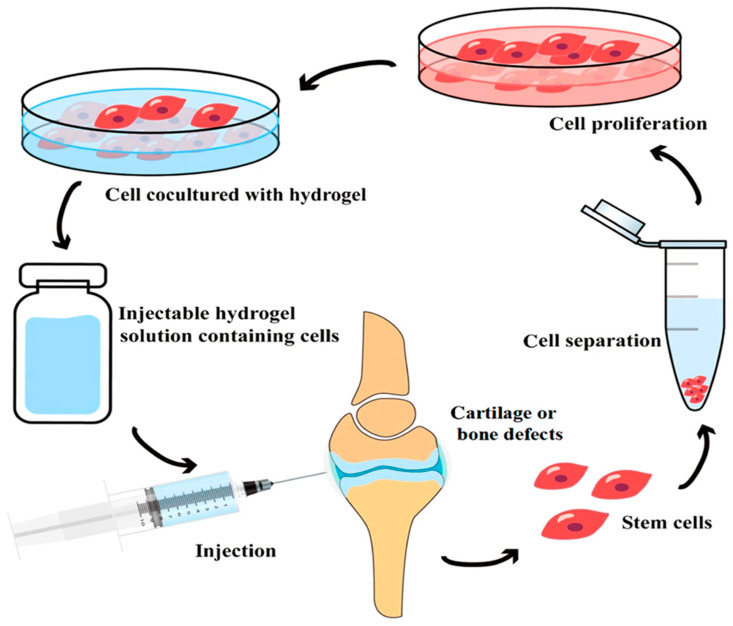
Schematic representation of an innovative application of injectable hydrogel for bone regeneration. Reprinted from Ref. [287].

The polymers are joined to form a wet, malleable, and porous structure. These hydrogels have the ability to convert electrical impulses effectively. Syk 2006 attributes this to the polymer backbone’s inherent electrical conductivity. Furthermore, these hydrogels possess mechanical properties comparable to those of biological tissues because of their high water content and hydrophilic nature, which enhances their compatibility with biological systems [284,286]. Figure 7 concisely demonstrates the primary benefits of using polymer/organic based injectable hydrogel system in medicine and especially regenerative medicine, such as:

(1) Minimally Invasive Administration: The liquid formulation of hydrogel can be injected directly into the irregular defect location via a tiny incision, eliminating the need for open surgery and enhancing patient recovery.
(2) The hydrogel serves as a transient. Upon solidification (in situ gelation), it wraps the stem cells, furnishing them with a conducive environment for attachment, proliferation, and differentiation into the targeted cell type (e.g., cartilage or bone cells).
(3) Patient-Specific Therapy: This approach often employs the patient’s own stem cells (autologous cells) through hydrogel, therefore minimizing the danger of immunological rejection and facilitating spontaneous recovery.

Hydrogel technology signifies a significant transition from pre-formed, inflexible scaffolds to dynamic, cell-transporting liquid systems that solidify inside the body, effectively filling defects and actively facilitating tissue regeneration [287].

Additionally, hydrogel chemicals allow for easier drug delivery and better blending with biological tissues, which makes them especially good for uses like heart tissue engineering, brain connections, and healing medicine. Injectable conductive hydrogels can be customized to meet the specific needs of different biological electronic systems and treatments by changing the makeup of the polymers, the density of the connections between them, and adding extra functional parts. Such customization makes it possible to regulate the mechanical, physiological, and electrical properties of the hydrogels [288].

#### 6.2.2. Organic Nanowires

Nanowires are nanostructures that are characterized by a high aspect ratio and a single dimension. Their typical sizes range from a few nanometers to several hundred nanometers, and their lengths may exceed several micrometers [289,290,291,292]. The ability of organic semiconductor nanowires to form intimate, highly conductive bonds with biological tissues enables efficient electrical signal stimulation, transmission, and recording [292]. They are thus highly suitable for a wide range of applications in organic bioelectronics [293].

(1) Organic nanowires’ unique characteristics, such as their large surface area and electrically tunable characteristics, make them ideal for creating sensitive and selective biosensors. With the use of these biosensors, physiological indices and biomolecular interactions may be continuously monitored in real time [294].
(2) Drug Delivery: Nanowires may be modified to improve the effectiveness and reduce the adverse effects of pharmacological therapies by using medicinal chemicals and nanocarriers for precise and regulated drug delivery [295].
(3) Tissue Engineering: By integrating into scaffolds, organic semiconductor nanowires stimulate tissue regeneration by supplying electrical stimuli and assisting cells in developing and specializing [296].
(4) Bioelectronic Implants: Devices based on organic nanowires may be created to be implanted with the least amount of intrusion. Through tight, stable, and long-lasting interactions with biological systems, these devices enable a variety of diagnostic and therapeutic applications.

#### 6.2.3. Organic Nanotube

Organic semiconductor-based nanotubes are now being researched for a variety of applications, such as medication delivery, sensors, catalysts, optical devices, and biological applications. Lipids, peptides, DNA nanotubes, polymer-ONT conjugates, and hydrogels are required for these applications [297].

### 6.3. System Integration and Interfaces

Organic bioelectronic interfaces are one of the main topics of bioelectronics research. Their primary goal is to create seamless and compatible connections between biological systems and technological equipment to facilitate integration and two-way communication [298,299,300,301].

## 7. Electrode-Tissue and Electrode-Cell Interactions

Electrodes are crucial in many biomedical applications because they establish electrical interactions with biological processes. These include brain interface, cardiac pacing, and biosensory. significantly, these electrode-based devices’ compatibility, stability, and usefulness are Most determined by their interactions with the biological environment, which includes cells and tissues [300,302]. Both Faradaic reactions, which involve the movement of electrons between the electrode and the organism, and non-Faradaic processes, like charging and layer formation at the interface, can lead to charge transfer at the electrode-tissue interface.

The surrounding tissue may be impacted by pH [303], reactive oxygen species [304], and metallic ion ejection [95,96,302] generated by electrochemical activities at the contact [305,306]. Carefully regulating these processes is necessary to reduce any adverse effects on the tissue. When an electrode is placed in the body, the body may react by forming scar tissue around it. The electrode’s encapsulation may hinder the mechanical and electrical connection between the electrode and the targeted tissue, jeopardizing the device’s functionality [302,307,308].

The electrode’s surface characteristics, such as its topography, chemistry, and charge, may have an impact on the form, adhesion, and growth of cells that come into contact with it. By enhancing these surface properties, it is possible to optimize the interactions among cells and electrodes and, therefore, the integration of the electrodes with their supporting cellular environment.

Electrodes can affect how cells, like heart cells and nerve cells, behave electrically by changing how easily they can get excited and how their electrical charge moves along their membranes. Understanding these interactions is essential for creating electrodes that can efficiently attach to and capture data from excited cells [309].

Inserting an electrode into the body may trigger a foreign body response, leading to the formation of fibrotic tissue around it. Thus, the encapsulation technique may hinder the electrode’s mechanical and electrical connection to the tissue it is targeting, thereby impacting the device’s operation [310].

The adherence, growth, and morphology of cells that come into contact with the electrode may be impacted by the electrode’s surface characteristics, such as topography, chemical reactions, and charge. Cells may interact with the electrode and become more integrated with the local cellular environment if certain surface characteristics are optimized [311,312].

The literature documents numerous methods to improve the interactions among electrodes, tissues, and cells. The contact between electrodes and tissues or cells is enhanced by surface modification, coatings, and bioactive substances such as hydrogels or conductive polymers. This process facilitates the integration of tissues and improves signal transmission. The electrode’s flexibility and miniaturization may enhance the mechanical engagement with the target tissue and reduce the foreign body response. Thus, this integration enhances the long-term stability and effectiveness of electrode-dependent devices. Furthermore, the development of electrodes that mimic the physical, chemical, and electrical properties of natural tissue, or biomimetic design, has the potential to enhance the overall compatibility of electrode-dependent devices and thereby boost integration [313,314,315].

The creation of reliable and efficient electrode-based biomedical devices depends on knowledge of the complex interactions that exist between electrodes and their immediate biological environments. The biological compatibility as well as efficiency of these electrode-tissue and electrode-cell interfaces will be enhanced by ongoing research and development in the fields of materials, surface technology, and device design [316].

**Figure 8:**
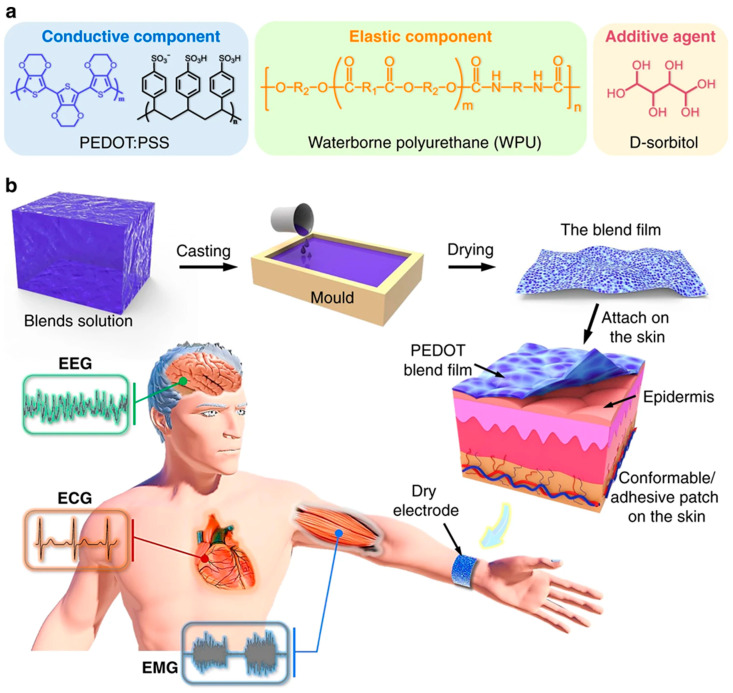
Fabrication and implementation of a conductive, elastic organic/polymer hybrid bioelectrode (**a**) The composition of the blend film includes the conducting polymer PEDOT:PSS, an elastic waterborne polyurethane (WPU) component, and the additive D-sorbitol. (**b**) A diagram depicting the manufacturing process, in which the mix solution is poured into a mold and then dried to create a film. The resultant electrode is seen affixed to the epidermis, serving as a dry electrode for the collection of electrophysiological signals, including electroencephalography (EEG) and electrocardiography (ECG). Reprinted from Ref. [317].

Electrodes, shown in Figure 8, can affect how cells, like neurons and heart cells, communicate electrically by changing how easily they can be stimulated, which alters the charge moving across the cell membrane and affects ion transport. Knowing how these interactions work is important for creating electrodes that can effectively connect to and either stimulate or collect signals from active cells. This figure clarifies the design philosophy and manufacturing procedure for an advanced bioelectronic electrode, emphasizing the crucial significance of PEDOT:PSS as a conductive electrode element. The material is significant due to its superior electrical conductivity and distinctive capacity to carry both ions and electrons, allowing an effective, low-impedance contact with the ionic environment of human skin for precise signal capture, such as electroencephalography (EEG). To address the intrinsic mechanical limitations of pure PEDOT:PSS, specifically its brittleness, it is combined with an elastic component, waterborne polyurethane (WPU), which imparts crucial stretchability and flexibility, enabling the composite film to adapt seamlessly to the dynamic contours of the skin. Additionally, the addition D-sorbitol functions as a supplementary dopant, augmenting the conductivity of the PEDOT:PSS network in the resulting mixture. The procedure entails pouring the aqueous solution into a mold and then drying it to create a solid, self-supporting film. This final product exemplifies a crucial paradigm in soft bioelectronics: by merging the electrical capabilities of PEDOT:PSS with the mechanical attributes of an elastomer, a novel class of devices is developed that integrates effortlessly with the human body, guaranteeing comfortable, prolonged wear and dependable monitoring of biological signals through stable, conformal contact even during motion [317].

### 7.1. Methods to Improve Coupling and Signal Transduction

The coupling and signal transmission in organic bioelectronic interfaces may be strengthened by a number of strategies that enhance the interaction and communication between biological components and organic bioelectronic devices [318,319]. Here are some crucial strategies:

#### 7.1.1. Biomimetic Construction

An interface that closely mimics the extracellular matrix and cellular environment would enhance signal transmission and the bond between organic bioelectronic conductors and tissues. Using bioactive substances that improve cell adhesion, proliferation, and differentiation at the interface is one practical approach. These compounds might be growth factors or peptides that promote cell adhesion. One effective method to improve connectivity to cells and signal transmissions is to replace the mechanical and topographical features of the natural tissue. Scientists can enhance the effectiveness, compatibility with living things, and outcomes of bioelectronic devices in medical settings by creating interfaces that mimic biological structures and exhibit biological activity, allowing for more engagement with biological systems [42,320,321].

Biocompatible conductive materials are another way to enhance signal transduction and connection in organic bioelectronics. Because of their desirable properties, such as electric conductivity, biocompatibility, and functionalize ability, organic conducting polymers like PEDOT and polypyrrole are well suited for integration into biological systems [104,322,323].

Signal transmission and the connection between the biological and electronic components may be greatly improved by including nanostructured compounds into the organic bioelectronic interface. Using certain nanostructured materials to increase the interface’s surface area may enhance interactions and provide more efficient electrical signal transmission. The characteristics and nanoscale topography of these nanostructured components are quite similar to those of the natural cellular environment. This similarity enhances cellular adhesion, growth, and coordination at the bioelectronic interface. Organic bioelectronic devices may provide improved communication with biological systems via nanostructures, hence enhancing biocompatibility and efficiency [324,325].

#### 7.1.2. Enzymatic and Electrochemical Coupling

Nano interface for organic bioelectronics efficiently transfer charge across biological and electronic components via enzymatic and electrochemical interaction. This technique makes it easier to incorporate biological elements, such enzymes, into electrical circuits, opening up new applications for biosensing and biomedical electronics [326,327]

#### 7.1.3. Neuromodulation Techniques

Nano interface of organic bioelectronics can allow advanced neuromodulation methods, providing more accurate monitoring and control of brain function [328]. Bidirectional communication may be formed by the integration of organic electrical nanostructured interfaces with brain tissues, opening up possibilities for the treatment of brain diseases and enhancing human–machine interactions. The nervous system may be able to communicate with these natural bioelectronic systems at the cellular level. By enabling very precise and intricate neural stimulation and recording, this outperforms earlier capabilities [329,330,331].

#### 7.1.4. Dynamic and Adaptive Interfaces

The design of interface that can respond to changes in the biological environment may be revolutionized by organic biological electronic at the nano interface [332]. By using the unique properties of organic electronic materials, such as flexibility, conformability, and biocompatibility, these adaptive interfaces enhance the relationships between electronics and living tissues, resulting in more responsive and seamless experiences. These interfaces may dynamically alter their properties via the use of stimuli-responsive components, improving signal transmission and lowering adverse bodily effects. Long-term, reliable, and accurate bioelectronic interactions are therefore made possible [333,334].

### 7.2. Surface Modifications and Functionalization for Improved Biocompatibility

The compatible nature of electrodes with living things is essential for the effective incorporation of organic bioelectronic systems into biological tissues. Surface alterations and functionalizing techniques may enhance the interaction between these electrode materials and the biological environment [48,66,335].

One solution to the issues of non-specific protein absorption and cell adhesion is to immobilize hydrophilic polymers, such as polyethylene glycol (PEG), which may also help avoid adverse foreign body responses. Furthermore, certain cell types’ incorporation and signal transduction may be enhanced by the addition of growth factors or cell-adhesive peptides to the electrode surface. This encourages focused, targeted interactions. Additionally, using conductive materials, such poly(3,4-ethylenedioxythiophene) (PEDOT), may enhance the electrical connection between biological tissue and organic electrodes, boosting the device’s overall effectiveness and charge transfer efficiency. Researchers can enhance biocompatibility and optimize the interaction between biological systems and organic bioelectronic materials by carefully designing and functionalizing electrode surfaces [314,336].

## 8. Achievements of Organic Bioelectronics

Thanks to recent developments, major milestones in organic bioelectronics have already been reached; still, great work is needed to reach the final goals. These technical innovations have enabled the creation of electronic devices with characteristics of flexibility, stretchability, and fit with biological tissues. Successful integration of these organic electrical devices with the human body enables their use in drug delivery, brain interfacing, cardiac monitoring, and several other forms of medication administration. Advances in organic bioelectronics have opened doors for tailored, flexible, minimally invasive healthcare solutions that might greatly raise human capabilities and improve quality of living. Here only few of these successes are covered [31,337].

### 8.1. Neural Interfaces and Brain–Computer Interactions

Advancement of advanced neural interfaces and brain–computer interaction (BCI) technologies depends greatly on organic bioelectronics. Because of their special properties—biocompatibility, flexibility, and interaction with biological systems—organic electronic materials are quite appropriate for use in this field. Electronic devices interacting with neurons to address vision loss, [338], hearing loss [339,340]. Recent prosthetic developments aiming at the central nervous system have focused on seizure disorders [341,342], depressed disorders [343], urinary tract infections [344,345] and pain management [346,347].

One important use of organic bioelectronics in neural interfaces is the development of electrodes and sensors displaying remarkable recording and triggering capability for brain activity. Conductive polymers, such PEDOT (poly(3,4-ethylenedioxythiophene), can be altered to show electrical and mechanical traits [348] akin to those of neural tissue. This allows a close and continuous integration with the brain and nervous system. Action potentials and local field potentials are among the high-fidelity electrophysiological signals these organic electrodes can record. This helps to gather a great volume of data about cerebral activity and communication [336].

Furthermore used for therapeutic purposes in the domains of neurology and neurological rehabilitation could be the capacity of organic electronic materials to provide electrical stimulus. By means of organic electronic devices in close proximity to neural tissues to modulate neural activity, persons with neurological disabilities can be treated for conditions including Parkinson’s disease [349,350], chronic pain [351], and the restoration of sensory functions [352], namely vision or hearing [353,354].

Brain-Computer interfaces (BCIs) for recording and stimulation are developing organically under organic bioelectronics. Users of this technology can run prosthetic appendages or applications using their neurological activity. These systems are made to fit the human body perfectly and offer a great degree of durability, so reducing the possibility of causing any injury or damage. Organic bioelectronics’ adaptability helps to incorporate closed-loop control, signal processing, and brain interface devices among other things. For those with neurological diseases or disabilities, this offers chances to improve understanding of the brain, therapeutic treatments, and quality of life [355,356,357].

### 8.2. Wearable and Implantable Biomedical Devices

Organic electronic materials have proven very useful for wearable and implantable biomedical devices due to their remarkable flexibility, biocompatibility, and capacity to fit numerous shapes [358]. These devices have the amazing capacity to precisely suit the human body, opening up a wide range of potential medical applications. Organic electronics may be used by wearable technology to create discrete and enjoyable monitoring systems. These platforms may include organic electrochemical devices and electrodes. Organic electronics are ideal candidates for seamless interaction with living tissues in implanted biomedical devices. This facilitates the development of neural interfaces and tissue regeneration. Developing closed-loop systems that can reproduce and monitor biological processes for therapeutic purposes requires a thorough understanding of electrical systems. Organic bioelectronics are very versatile and make it simple to integrate several features into a single device. Thus, very complex and adaptable biomedical systems are developed that may easily satisfy the constantly changing physiological requirements [359].

### 8.3. Environmental Monitoring and Remediation

Organic bioelectronic devices might be very beneficial for environmental monitoring and remedial applications. They are well suited for on-site environmental monitoring and corrective action because of their unique characteristics, which include their capacity to interact with living things effectively, sensitivity to environmental changes, and flexibility [360].

Several environmental contaminants and poisons might be identified and measured using organic electronic sensors for environmental monitoring. It will be possible to collect data on the condition of the surrounding ecosystem in real time if these sensors are developed to detect certain chemical species, pH levels, or additional atmospheric parameters. Organic electrochemical transistors may be used to detect heavy metals, herbicides, and other dangerous substances in water. The flexibility and conformability of organic materials allow sensors to be incorporated into implantable or wearable devices, allowing for continuous ambient monitoring in remote or challenging-to-reach locations [16,361].

Additionally, organic bioelectronics might help with environmental cleaning operations. Because of their inherent conductivity and redox-active characteristics, conductive polymers may aid in the electrochemical breakdown or transformation of environmental contaminants. These substances might be used as electrodes in electrochemical remediation systems to aid in the oxidation or reduction in contaminants and their subsequent removal from the environment. Furthermore, the compatibility of organic materials with living things enables the creation of biohybrid systems. These self-sustaining platforms combine live things, including bacteria or microbes, with organic electrical components for restorative purposes [362,363].

Organic bioelectronics has considerable potential as a solution to solve a number of environmental issues, such as process optimization, pollution detection and monitoring, and on-site treatment. They are crucial in promoting a more sustainable and ecologically friendly future because of their ability to adapt to their environment [57,364].

### 8.4. Tissue Engineering and Regenerative Medicine

Organic bioelectronic materials have recently been identified as a very promising platform for the application of tissue engineering and regenerative medicine. A number of aspects of tissue regeneration and restoration might be enhanced and made possible by these materials’ unique properties [365].

One important use of organic electronic frameworks is the establishment of a biocompatible and appropriate environment for cell proliferation and tissue formation. Conducting polymers, such PEDOT (poly(3,4-ethylenedioxythiophene)) [366], may be used in 3D scaffold designs to enhance ion and electrical impulse flow. This may have an impact on cellular activity and enhance tissue integration. This may be particularly beneficial for the regeneration of electrically active tissues, such as cardiac, neural, and muscular tissues. Electrical impulses may be sent and provided to enhance tissue structure, cell differentiation, and functional restoration [367].

To provide continuous monitoring and control, organic bioelectronics may also be used in conjunction with other tissue engineering methods. Physiological characteristics like pH, oxygen levels, and electrical activity may be continuously monitored with the use of organic electrodes and sensors embedded in tissue structures. This helps to improve cultural circumstances and enhances the assessment of tissue maturity [17]. The development of intelligent, reactive biomaterials that can adapt to the needs of the healing tissue may be aided by sensing capabilities [368].

Additionally, biological activity may be controlled and enhanced by organic bioelectronic devices acting as interfaces. Organic electrodes may be used to promote nerve regeneration, enhance the development of blood arteries in tissues, and regulate the differentiation of stem cells, among other regenerative processes. Additionally, electrical or electrochemical signals may be used. Exact control of bioelectronic stimuli is essential for guiding the complex chain of events that occur during tissue healing and regeneration [283].

The unique properties of organic electronic materials may be used by researchers in the fields of tissue engineering and regenerative medicine to develop innovative concepts that facilitate the body’s natural ability to renew itself. This leads to better patient outcomes and more sophisticated treatment options.

### 8.5. Drug Delivery

Organic bioelectronics have a lot of potential for application in medicine delivery systems. Low-molecular-weight organic semiconductors have special properties that make them suitable for a variety of drug delivery methods [369]. Organic bioelectronics have the potential to greatly enhance medication delivery across a number of crucial processes.

It is possible to regulate and respond with medication release by designing organic semiconductors that can react to certain stimuli, such as pH, temperature, or the presence of specific proteins. Controlling the time and place of therapeutic agent delivery may be made easier by a quick and efficient reaction, which may enable the controlled and triggered release of medications. By using sensors made of organic semiconductors, the drug delivery system may also monitor the local environment and alter the release pattern in real-time to increase treatment effectiveness [370,371].

Drugs may be purposefully given to specific cells or tissues by modifying organic semiconductors with specific molecules, such as ligands, peptides, or antibodies. The focused strategy may improve the therapeutic index by promoting medication accumulation at the desired location and preventing unintended effects elsewhere. Combining organic semiconductor-made sensors might provide real-time medication administration monitoring and the response at the target area. This capability aids in the development of adaptable and personalized treatment programs [372].

It may be possible to intentionally create low-molecular-weight organic semiconductors that decompose spontaneously, allowing for the creation of short-term drug delivery systems that can be safely removed from the body after their intended medicinal usage. This strategy may be especially helpful when short-term drug administration is necessary, such as when managing post-operative pain or wound healing. It reduces the need to remove the drug-delivery device surgically [66,373,374].

When organic semiconductors are incorporated into bioelectronic interfaces, such wearable patches or implanted devices, they may help make it possible to continuously monitor physiological data and dispense drugs based on certain situations or triggers. This integration has the potential to improve therapeutic outcomes and reduce the likelihood of side effects by utilizing closed-loop drug delivery systems that can automatically adjust medication administration based on the patient’s needs [375,376].

Organic semiconductors may have combined effects with other organic bioelectronic devices, such as medication delivery systems, biosensors, or brain interfaces. Research in organic bioelectronics and its convergence with drug delivery systems shows great promise for the development of innovative and sophisticated therapeutic solutions, so offering enhanced effectiveness, safety, and patient-centered care [377]. For instance, the ability to monitor physiological data or neuronal activity could provide useful information for the medication delivery process [378,379], enabling more specialized and flexible treatment approaches.

### 8.6. Biodegradable Organic Bioelectronics

Organic bioelectronics provide substantial potential for the advancement of medical applications due to their superior biocompatibility, capacity to communicate with biological tissues, and supply of sustainable solutions. The application of biodegradable materials is essential to achieving that potential, as encapsulated by their primary benefits:

(1) Biocompatibility and Safe Integration: Most natural polymers (e.g., polylactic acid, chitosan, gelatin) and biodegradable synthetic polymers (BSPs) have favorable tolerance within biological systems [93,148]. Their progressive degradation by the body modulates the immune response, resulting in less inflammation and enhanced tissue integration compared to conventional non-biodegradable implants, which commonly activate a foreign body reaction [380,381,382].
(2) Transitory Functionality and the Removal of Surgical Extraction: A major advantage is the ability to fabricate transitory devices that execute a necessary function for a designated duration before safely dissolving. This obviates the need for a further surgical intervention to extract the implant, therefore reducing long-term problems and patient burden [383]. This is especially relevant for applications such short-term brain interfaces, biodegradable physiological sensors, and programmable drug delivery systems [75].
(3) Inherent Sustainability: The biodegradable characteristics of these materials guarantee their decomposition and integration post-use, according to overarching objectives of environmental sustainability and eco-friendly medical technology [124].

## 9. Challenges

As the science of organic bioelectronics advances toward therapeutic applications, it is imperative to recognize and address the ethical and regulatory concerns associated with its development and usage. Assuring the safety, efficacy, and responsible usage of organic bioelectronic devices must be given high attention in order to build public trust and promote their widespread use in the healthcare industry [62,384]. The convergence of organic electronics, biology, and medicine has led to significant advancements in organic bioelectronics. The achievement of long-term biocompatibility and biostability of organic electronic materials is one of the primary limitations that must be overcome if we are to fully use the benefits offered by this interdisciplinary approach [40].

(1) The seamless interaction with biological systems and scalable production are the limitations of seldom utilized organic bioelectronic devices from lab to clinic [385].
(2) Another restriction is the therapeutic use of organic bioelectronic devices brought on by moral and legal issues. Clinical application may be slowed down by intricate regulatory processes, approvals, data protection, informed consent, and accessibility concerns [386].
(3) The vocabulary, methods, and objectives of biology, medicine, and organic electronics may be outside the scope of effective communication. Collaboration is essential for information exchange and research goal alignment [387].
(4) High-performing organic bioelectronic devices may have issues with stability, inability to match silicon-based electronics’ capabilities, and reduced mobility of charge carriers in organic electronics [388].

The allocation of enough resources, technical advancements, harmonization of regulations, and ongoing interdisciplinary collaboration are all necessary to overcome these constraints and make it possible to conduct research and produce organic bioelectronic solutions. By tackling these problems, organic bioelectronics may reach its full potential and provide innovative medical solutions that bridge the gap between the biological and electrical realms.

## 10. Future Perspective

Organic bioelectronics is a rapidly evolving field that has the potential to revolutionize biology, healthcare, and human–machine interactions. Utilizing the special qualities of organic materials, such as their flexibility, biocompatibility, and ability to interact with biological systems, these technologies provide innovative solutions that may seamlessly interact with the human body and the environment. Organic bioelectronics has enormous potential and is poised to bring about significant changes, as seen by the ongoing advancements in this area of study and development [23].

The ongoing progress in the field of organic materials research is the foundation of organic bioelectronics. Researchers are examining a broad range of organic semiconductors, conductive polymers, and biomolecular materials with advantageous mechanical, optical, and electrical characteristics for a range of bioelectronic applications. The performance and reliability of organic bioelectronic devices will be enhanced by the discovery of novel organic materials with improved stability, charge transport properties, and biocompatibility [41,389,390].

The capacity of organic bioelectronics to adapt to intricate, curved, and flexible biological surfaces is one of its key benefits. Polymers, hydrogels, and bioinspired materials are examples of flexible and elastic substrate materials that have enabled the development of organic bioelectronic devices with aesthetically beautiful and subtle interfaces that easily integrate with the human body. These adaptable devices may be used for a number of purposes, such as providing body-based medicinal treatments and monitoring physical health. This creates new prospects for customized healthcare [391].

The main goal of organic bioelectronics is complete integration with biological systems so that the devices may develop in a way that benefits both parties. Researchers are trying to find methods to make organic materials more durable and able to interact with live tissues in a manner that will enable them to be implanted in the body for extended periods of time while maintaining their original function. The development of biohybrid systems, which integrate organic electronics with living cells or tissues, is also very promising for use in regenerative medicine [392], brain interfaces [393], and intelligent prostheses [394].

The unique capacity to combine many functionalities onto a single substrate is provided by organic bioelectronics. Researchers are creating organic bioelectronic devices that may be used for sensing, actuation, data processing, and medication administration by using the versatility of organic materials. High levels of multifunctional integration may result in the development of intelligent, closed-loop systems that are able to dynamically adjust to the user’s physiological requirements and environmental circumstances, enabling distinctive and autonomous healthcare solutions [40,395].

One of the main obstacles to the widespread use of organic bioelectronics has been the need to develop scalable and affordable production processes. Advances in additive manufacturing methods, like 3D printing, screen printing, and inkjet printing, have made it possible to build organic bioelectronic devices in a timely and customized manner. The availability and financial sustainability of organic bioelectronics may also be increased by combining automated and high-throughput manufacturing processes, which may greatly increase efficiency and reduce production costs [337,396,397].

The number of successful clinical trials and regulatory clearances for organic bioelectronics is increasing as the field develops. Organic bioelectronic technologies, such as wearable health monitoring and implanted brain interfaces, are being used from the lab to real-world applications. In the clinical setting, these instruments are fulfilling requirements and enhancing patient results. Additionally, organic bioelectronics show potential not just in the medical field but also in other fields including environmental monitoring, human–machine interaction, and smart agriculture [23,398].

The potential of organic bioelectronics is enormous. Researchers and engineers are combining the special qualities of organic materials with modern production processes, leading to advancements in biomedical and technological breakthroughs. As the field continues to advance, organic bioelectronic devices need to become increasingly integrated with the human body. This integration will provide greater options for human–machine interaction, improved quality of life, and personalized healthcare [16,399].

Conjugated polymers, particularly PEDOT:PSS, and carbon-based materials, including graphene and carbon nanotubes, have the most promise for long-term clinical use among the many materials assessed. The exceptional electrical conductivity, superior biocompatibility, ease of manufacturing, and proven efficacy of PEDOT:PSS in many devices, such as OECTs and brain interfaces, distinguish it from its competitors. A disadvantage is its susceptibility to oxidation under physiological conditions and its considerable mechanical fragility. The exceptional electrical properties, mechanical strength, and extensive surface areas of graphene and carbon nanotubes facilitate durable neural interfaces and highly sensitive biosensors; nonetheless, challenges regarding long-term biostability, potential cytotoxicity, and intricate functionalization must be resolved. Natural polymers such as silk and chitosan have excellent biodegradability and biocompatibility; yet, their inadequate electrical conductivity precludes their use in high-performance bioelectronics. Hybrid and composite materials including organic conductors with enhanced stability and biocompatibility seem to be the most promising option for future clinical applications.

The future direction of organic bioelectronics will be determined by meeting, quantitative benchmarks that confirm its shift from laboratory potential to clinical and commercial application. The field must exhibit closed-loop neural interfaces that can detect epileptic seizures with a latency of less than 50 ms and provide therapeutic stimulation with a charge injection limit exceeding 5 mC cm^−2^, as outlined in current translational studies for drug-resistant epilepsy. In regenerative medicine, bioelectronic scaffolds must demonstrate efficacy in clinical trials by achieving a statistically significant increase, such as a 40% enhancement in axon regeneration rates compared to controls when utilizing electrical stimulation through PEDOT-based conduits in peripheral nerve repair. For broad implementation in diagnostics, wearable OECT-based biosensors need to provide continuous, multi-analyte monitoring (e.g., simultaneous tracking of cortisol and glucose) with a coefficient of variation of less than 5% over 72 h during ambulatory human trials. Additionally, scalable manufacturing will be demonstrated through the implementation of roll-to-roll printing to create organic electrodes, achieving a device-to-device performance variation of less than 3% and a production cost of under $0.10 per cm^2^. Achieving these specific metrics will serve as the definitive assessment of the field’s success, transitioning from theoretical potential to providing validated solutions that satisfy rigorous clinical and market requirements.

## 11. Interdisciplinary Collaborations

Organic bioelectronics intersects with biology, electrical engineering, materials science, and medicine, necessitating the involvement of multidisciplinary teams for the advancement of the field. The integration of biological knowledge and clinical insights is essential for the development of high-performance organic semiconductors, facilitating the creation of devices such as brain user interfaces, biological sensors, and bioelectronic actuators. Engineers contribute to the design of circuits, microfabrication, and device integration, while materials scientists specialize in modifying polymer chemistry, morphology, and stability. Physicians facilitate the translation of research to meet patient needs, while biologists and neuroscientists ensure the compatibility of these devices with biological systems [400,401].

Developing neural interfaces for deep brain stimulation in Parkinson’s disease using PEDOT:PSS is a prime example of how multidisciplinary cooperation accelerates clinical translation. Improved conductivity and flexibility were achieved by materials scientists, microelectrode arrays were efficiently built by engineers, stimulation locations were optimized by neuroscientists, and patient-specific implantation was supervised by physicians. Additionally, regulatory specialists oversaw the procedures for safety and approval. Collaboratively, these interdisciplinary teams overcame material, functional, and clinical obstacles to bring PEDOT:PSS devices to the next level, from laboratory prototypes to first-in-human trials [402,403,404].

The development of wearable devices utilizing biosensors based on organic electrochemical transistors (OECTs) represents a significant advancement in diabetes treatment. Chemists collaborated with engineers to enhance the sensitivity and selectivity of channel materials by developing low-power circuitry for real-time reading and integrating sensors onto flexible substrates. Data scientists have applied machine learning techniques for the monitoring and early detection of metabolic issues. Diabetes specialists established the trial methodology, patient usability requirements, and clinically relevant biomarkers. This collaborative effort enabled the integration of OECT biosensors into skin-mounted patches, facilitating continuous metabolite monitoring outside of hospital settings [405,406,407,408].

## 12. Conclusions

A breakthrough decade in medical and biosciences has begun with organic bioelectronics. The domain of organic bioelectronics has evolved from a fundamental concept into a robust, multidisciplinary discipline, demonstrating significant potential via a diverse array of applications, including wearable sensors, tailored drug delivery systems, organic electrochemical transistors, and brain interfaces. The intrinsic advantages of organic materials, including their exceptional biocompatibility, mechanical flexibility, and versatile ionic-electronic transduction, provide a unique foundation for establishing seamless interfaces with the intricate dynamics of biological systems.

Nonetheless, significant barriers must be overcome to advance from an innovative prototype to clinical and commercial viability. The careful design of devices for enhanced long-term performance, stability, and reliability within biological environments is a critical task that remains ahead. The development of reproducible, scalable fabrication techniques is essential for widespread acceptance. It is essential to rigorously evaluate the ethical and legal frameworks that will ensure the appropriate development and use of these powerful technologies as they advance.

Three significant advancements will influence the future of organic bioelectronics: the discovery of novel, multifunctional materials; the innovative design of more intricate and compact devices; and, most importantly, enhanced collaboration within the fields of materials science, engineering, biology, and medicine. The field is poised to go beyond mere observation to closed-loop therapeutic systems capable of diagnosing, monitoring, and treating illnesses with unprecedented precision with the adoption of this convergent methodology.

In summary, organic bioelectronics are a gateway to a new paradigm where the boundaries between technology and biological sciences are blurring, making it more than simply an engineering job. Further study in the area may redefine human–machine symbiosis, revolutionize healthcare, and enhance our understanding and control of fundamental life processes. The integration of biology with these advanced, adaptive systems suggests an era in which technology will more seamlessly link, heal, and augment humans than ever.

## Figures and Tables

**Table 1:** A framework for the classification of functional materials and devices, emphasizing their application fields, fundamental characteristics, and prevalent material groups.

Domain of Applications	Examples	Essential Required Attributes	Common Materials/Device Categories	References
Neural Interfaces	Cortical recording, Deep Brain Stimulation, Spinal cord stimulation, Peripheral nerve interfaces, Vagus nerve stimulation	Biocompatibility, Low Impedance, Conformability, Chronic Stability, Charge Injection Capacity	Organic nanocolloids, PEDOT:PSS, PPy-based electrodes; OECTs; Soft hydrogel coatings, CNT	[73,74]
Implantable Sensors	Glucose monitoring, metabolite sensing, pH sensors	Biocompatibility, Selectivity, Sensitivity, Stability in biofluid, Miniaturization	Enzyme-functionalized OECTs; PANI/Ppy-based amperometric sensors, Poly(glycerol sebacate) (PGS) based dielectric sensors	[75,76]
Tissue Engineering Scaffolds	Cardiac patches, Nerve guidance conduits, Bone regeneration, Skeletal muscle regeneration	Biocompatibility, Biodegradability (often), Electroactivity, Appropriate Young’s Modulus (to match tissue), Porosity	modified polyurethane, PPy, PEDOT, PANI composites with PLGA, collagen, chitosan, Alginate (sodium), Silk fibroin	[77]
Wearable Sensors	Epidermal ECG/EEG, sweat biosensors	Flexibility/Stretchability, Comfort, Stability under deformation, Sensitivity	PEDOT:PSS/elastomer blends; CNT/polymer composites; Screen-printed OECTs, Polypyrrole (PPy)	[78]
Optogenetic Devices	OLED-based neural stimulators	Biocompatibility, High Luminescence Efficiency, Flexibility, Spatial Resolution, sensitive to local electric fields	Flexible OLEDs; Light-emitting polymers	[79]
Drug Delivery Systems	Iontophoretic patches, responsive release systems	Biocompatibility, Stimuli-Responsiveness (redox, pH), Controllable Kinetics, Biodegradability (for implants)	Fullerence Drtivaties, Conducting polymer membranes (PPy, PEDOT); Hydrogel composites, Fluorescent Organic Nanoparticles	[80]
Bioelectronic Actuators	Artificial muscles, microfluidic pumps	Large Strain, Fast Response, Cyclical Stability, Low Operating Voltage	Conducting polymer actuators; Ionic polymer-metal composites (IPMCs)	[81,82]

**Table 3:** Applications of Conductive Polymers and Composites in Sensing and Biomedical Technologies: From Biosensors to Cancer Detection.

Organic Material	Electronic Device	Application	References
Polydopamine (PDA)	OECT	Cancer progression and aggressiveness biomarkers	[168]
PANI (membrane)	Interdigital	pH Sensor	[169]
PANI	Impedance	Humidity Sensor	[170]
PANI (nanopillars)	Electrode	pH Sensor	[161]
PEDOT:BTB	OECT	pH Sensor	[171]
PEDOT:PSS	OECT	Cl^−^ Sensor	[172]
PEDOT: Tosylate-polyamine	OECT	glucose biosensing in human urine samples	[173]
PEDOT:PSS	OECT	Na^+^, K^+^	[174]
PEDOT:PSS	OECT	Na^+^, K^+^, and pH Sensor	[175]
PEDOT:PSS	OECT	NH_4_^+^, and Ca^2+^	[176]
Manganese tetraphenylporphyrin (MnTPP)	OECT	Tyrosine	[177]
PEDOT: PSS	OECT	Electrochemical Compound Sensor	[178]
PEDOT: PSS	OECT	miRNA 21, cancer biomarker detectors	[179]
PANI	OECT	Electrochemical Compound Sensor	[180,181]
PANI	Impedimetric Sensor	Lactate	[182]
PANI@rGO	Amperometric Sensor	Glucose	[183]
PEDOT:PSS	OECT	EP, DA, AA	[184]
PEDOT:PSS	OECT	Cartisol	[185]
PEDOT:PSS	OECT	miRNA 21, Biological Interfacing applications	[186]
GO-doped PU@PEDOT	Nanofiber-Based Electronic Skin with Pressure, Strain sensor	Monitoring of human health and	[187]
Nanofibers		full-range motions	
MWCNT/carbon nanofiber/PEDOT:PSS	Electrospinning/drop casting	Muscular Actuator Sensor	[188]
PPY	Inkjet Strip	H_2_O_2_, Glucose Sensor	[189]
AgNW-PDMS	flexible and wearable electronic devices	Resistive strain sensor	[190]
AgNWs and Ecoflex	electronic capacitor devices	Wearable multifunctional sensors	[191]
Polydiacetylene-Polydimethylsiloxane		Chloroform Sensor	[192]
PMNT		DNA fluorescent sensor	
Aptamer-molecularly imprinted polymer (Apta-MIP)	molecularly imprinted polymer based electrochemical capacitance sensor	electrochemical sensor for the detection of bacteria	[193]
Graphene Oxide	OECT	Human cervical cancer (miRNA 21)	[194]
GCE/MWCNT/DOMIP s	OECT	serotonin (SE) detection	[195]
poly(benzimidazobenzophenanthroline)	OECT	on-skin detection of glucose, lactate, and	[196]
		uric acid	
PEDOT:PSS	OECT	Oxygen Sensor	[190]
Pentacene	OECT	Humidity Sensor	[197]
P3HT	OECT	Ion sensor (K^+^)	[198]
P3HT	ISFET	Na^+^, K^+^, Ca^2+^	[199]
PDTT	OFET	SO4^2−^	[200]
NiPC	OFET	Photosensor, humidity sensor	[201]
OND	OECT	Electrochemical Sensor	[202,203]
OND			[204]
PTAA	Dual Gate FET	pH Sensor	[205]
DDFTTF	OTFT	pH Sensor	[206]
DDFTTF	OTFT	TNT Sensor	[207]
α6T	OTFT	Glucose Sensor	[208]
DHα6T	OTFT	Lactic acid	[209]
CuPC	OTFT	Pyruvic acid	[210]

**Table 4:** Performance metrics for organic electrochemical transistors, highlighting materials parameters including threshold voltage (V_th), normalized transconductance (g_m,norm), switching time (τ_ON), on/off ratio (I_ON/I_OFF), and the product of mobility and volumetric capacitance (μC*). Entries are ordered by g_m,norm to facilitate comparison of current state-of-the-art performance.

Organic Material for OECT Channel	Vth [V]	gm_norm_ [S cm^−1^]	τ_ON_ [ms]	I_ON_/I_OFF_ (×10^3^)	μC [F cm^−1^ V^−1^ s^−1^]	Ref.
PEDOT:PSS (EG-P)	NA	5.26	NA	NA	100	[231]
p(gDPP-TT)	NA	2.5	NA	NA	125	[109]
p(gDPP-T2)	NA	7	NA	NA	342	[109]
BBLs	0.18	4.04	0.43	200	10.2	[232]
BBLn2	0.21	1.92	0.52	83	4.9	[232]
BBLis	0.27	0.617	0.89	2.9	1.94	[232]
PEDOT:PSS	NA	8	37	NA	NA	[233]
PEDOT:PSS	NA	7.14	NA	NA	NA	[234,235]
p(Cr-T2-OMe)	0.46	0.1	NA	NA	0.07	[236]
p(Cr-T2-Cp-EG)	0.32	0.31	24.6	NA	0.22	[236]
p(Cr-T2-Cp-EG)	0.3	0.02	6.2	NA	0.01	[236]
p(Cr-T2-Cp-EG)	0.33	0.01	12.5	NA	0.006	[236]
p(Cr-T2)	0.3	2.28	9.6	NA	1.29	[236]
p(Cr-T2)	0.24	0.63	7.5	NA	0.3	[236]
p(Cr-T2)	0.27	0.4	6.3	NA	0.2	[236]
p(Cr-T2)	0.37	0.15	12.7	NA	0.13	[236]
PEDOT:PSTFSILi100	NA	1.705	90	NA	NA	[237]
PEDOT:PSS	NA	1.755	49	NA	NA	[237]
p(gDPP-MeOT2)	NA	1.7	NA	NA	195	[238]
P90	0.24	0.009	41	1.9	0.0343	[239]
BBL	0.19	0.815	5.2	1.6	1.99	[239]
2DPP-OD-TEG	0.89	0.73	500	200	7	[240]
P(βNDI-βT2)	0.26	0.13	NA	1.4	0.06	[241]
p(C6-βNDI-βT2)	0.37	0.37	NA	2	0.16	[241]
p(C3-βNDI-βT2)	0.25	0.34	NA	2	0.13	[241]
BBL	0.21	0.359	900	6	NA	[242]
PgNgN	0.21	0.007	NA	1	0.037	[243]
PgNaN	0.37	0.212	127	10	0.662	[244]
P-90	0.26	0.21	NA	4	NA	[244]
P-75	0.29	0.141	NA	0.55	NA	[244]
P-50	0.36	0.067	NA	0.5	NA	[244]
P-100	0.25	0.204	NA	1.1	NA	[244]
P(βNDI-βT2)	0.28	0.1085	5	3.2	NA	[245]
P-90:TBAF(80%)	0.25	0.0833	NA	0.2	NA	[223]
P-90:TBAF(40%)	0.22	0.0905	24	1.2	NA	[223]
P-90:TBAF(00%)	0.25	0.0299	NA	0.17	NA	[223]
P-90	0.25	0.0113	NA	0.19	NA	[223]
P90, PFBT	0.29	0.0111	NA	NA	0.0008	[246]
C60-TEG	0.55	0.0146	80	25	7	[246]
p(NDI-T2-L2)	0.22	0.84	40	0.22	0.0046	[247]

## Data Availability

The data will be available on request.
